# Salient Object Detection in Optical Remote Sensing Images Based on Hierarchical Semantic Interaction

**DOI:** 10.3390/jimaging11120453

**Published:** 2025-12-17

**Authors:** Jingfan Xu, Qi Zhang, Jinwen Xing, Mingquan Zhou, Guohua Geng

**Affiliations:** 1 School of Journalism and Communication, Shaanxi Normal University, Xi’an 710062, China; jingfanxu@snnu.edu.cn; 2 School of Computer Science, Northwest University, Xi’an 710127, China; 202010302@stumail.nwu.edu.cn (Q.Z.); 202421904@stumail.nwu.edu.cn (J.X.)

**Keywords:** optical remote sensing images, salient object detection, hierarchical semantic interaction, channel adaptive enhancement, position-aware attention, multi-scale feature fusion

## Abstract

Existing salient object detection methods for optical remote sensing images still face certain limitations due to complex background variations, significant scale discrepancies among targets, severe background interference, and diverse topological structures. On the one hand, the feature transmission process often neglects the constraints and complementary effects of high-level features on low-level features, leading to insufficient feature interaction and weakened model representation. On the other hand, decoder architectures generally rely on simple cascaded structures, which fail to adequately exploit and utilize contextual information. To address these challenges, this study proposes a Hierarchical Semantic Interaction Module to enhance salient object detection performance in optical remote sensing scenarios. The module introduces foreground content modeling and a hierarchical semantic interaction mechanism within a multi-scale feature space, reinforcing the synergy and complementarity among features at different levels. This effectively highlights multi-scale and multi-type salient regions in complex backgrounds. Extensive experiments on multiple optical remote sensing datasets demonstrate the effectiveness of the proposed method. Specifically, on the EORSSD dataset, our full model integrating both CA and PA modules improves the max F-measure from 0.8826 to 0.9100 (↑2.74%), increases maxE from 0.9603 to 0.9727 (↑1.24%), and enhances the S-measure from 0.9026 to 0.9295 (↑2.69%) compared with the baseline. These results clearly demonstrate the effectiveness of the proposed modules and verify the robustness and strong generalization capability of our method in complex remote sensing scenarios.

## 1. Introduction

The theoretical foundation of Salient Object Detection (SOD) originates from the visual attention mechanism, whose core principle is to emulate the human visual system’s ability to prioritize regions with the most distinctive features within a scene, thereby accurately localizing areas of maximal visual contrast in an image. With continued research, the application of visual attention mechanisms has expanded considerably and is now widely integrated into various computer vision tasks, including image retrieval, object detection [1], image editing, image quality assessment, arc detection [2], and image classification. Over time, SOD has evolved into several research branches, such as Natural Scene Image SOD (NSI-SOD), Video SOD for dynamic sequences, and Remote Sensing Image SOD (RSI-SOD) for high-resolution remote sensing data, forming a comprehensive research framework. From a methodological perspective, SOD has progressed from early center-surround difference-based computational approaches to feature-driven traditional machine learning methods, and ultimately to deep learning-dominated architectures. The incorporation of deep learning [3] has not only substantially enhanced detection performance but also broadened the applicability of SOD to complex and multimodal environments [4].

Compared with natural scene images (NSIs), optical remote sensing images (RSIs) exhibit significant differences in object types, scale variations, illumination conditions, imaging perspectives [5], and background complexity. Consequently, directly transferring natural scene salient object detection (NSI-SOD) methods to remote sensing scenarios often yields suboptimal results. Nevertheless, the development of RSI-SOD has been largely inspired by NSI-SOD, particularly by convolutional neural network (CNN)-based approaches, which have provided valuable technical foundations for this field.

Among existing representative methods, LVNet enhances adaptability to multi-scale targets through multi-resolution feature fusion, while PDFNet further designs a five-branch, five-scale fusion architecture to achieve more comprehensive detection capability. EMFINet integrates multi-resolution inputs, edge supervision, and hybrid loss functions, yielding improved edge awareness but still exhibiting limitations under low-contrast and complex backgrounds. DAFNet employs shallow attention mechanisms to capture edge and texture information and deep features to capture semantic and positional cues; however, its interaction between high- and low-level features remains limited, and its decoder structure is relatively simple, leading to insufficient contextual information extraction.

Despite significant progress in RSI-SOD, existing studies still exhibit several common limitations. First, many methods struggle to handle complex and heterogeneous backgrounds, leading to incomplete or noisy saliency maps. Second, cross-level feature interactions are often insufficient, limiting the effective fusion of low-level spatial details with high-level semantic information. Third, decoder architectures are typically simple, which restricts the exploitation of contextual cues and multi-scale dependencies. Finally, the majority of approaches focus on specific datasets or scenarios, reducing their generalizability to diverse remote sensing conditions.Therefore, this study explicitly considers the complex background content inherent to optical remote sensing images. Moreover, to enhance the robustness of the proposed method, hierarchical semantic interaction modules are incorporated across multiple feature scales. Deploying these modules within a generic encoder–decoder backbone network is of particular significance. Accordingly, we propose a simple yet effective Hierarchical Semantic Interaction Network, which, benefiting from the backbone’s progressive inference process, effectively highlights salient regions of varying scales and object types while maintaining adaptability to challenging optical remote sensing scenarios.

The main contributions of this study are summarized as follows:

(1) A Hierarchical Semantic Interaction Network (HSIMNet) is proposed to explore the complementarity of multi-content feature representations in optical remote sensing images for salient region perception, enabling the joint utilization of both local and global image-level information.

(2) The Hierarchical Semantic Interaction Module is embedded across multiple feature scales within an encoder–decoder architecture, thereby seamlessly integrating the feature complementarity of the proposed module with the inference capability of the backbone network.

(3) The module incorporates an Efficient Channel Adaptive Enhancement component to adaptively adjust channel weights, capture global contextual information, and maintain computational efficiency. Combined with a Position-Aware Spatial Selection component that focuses on critical spatial regions, enhances spatial perception, and improves robustness, the two components synergistically integrate complementary information, enhance generalization, and improve detection accuracy and efficiency.

(4) Comprehensive experiments conducted on two RSI-SOD benchmark datasets demonstrate that the proposed method consistently outperforms state-of-the-art approaches across various evaluation metrics, validating the effectiveness of each component in the overall framework.

To provide a clear roadmap for readers, the remainder of this paper is organized as follows. Section 2 reviews related work on salient object detection in optical remote sensing images. Section 3 presents the proposed method, including the Hierarchical Semantic Interaction Module (HSIM) and the hybrid loss design. Section 4 describes the experimental setup, datasets, evaluation metrics, and presents the results and ablation studies. Finally, Section 5 concludes the paper and discusses potential directions for future research.

## 2. Related Work

### 2.1. Salient Object Detection in Natural Scenes

Traditional salient object detection methods primarily rely on handcrafted features extracted from natural images. However, their performance is often constrained by the limited representational capacity of these features. With the advancement of deep learning, approaches based on Convolutional Neural Networks [6] and Non-Local Structured Salient Object Detection (NL-SOD) have significantly improved detection accuracy. These methods typically employ various feature processing strategies—such as multi-scale feature interaction, feature suppression and balancing, edge-aware fusion, and context-aware aggregation—to more effectively capture salient information. Furthermore, to enhance model performance, recent studies have integrated techniques such as deep supervision, attention mechanisms, recurrent structures, generative adversarial networks (GANs), and adversarial learning [7]. Among representative works, Zhou et al. [8] proposed a semi-supervised framework that generates pseudo-labels via a boundary module and iteratively refines the saliency maps using a linear feedback mechanism. Qin et al. [9] developed an end-to-end network leveraging feature refinement and boundary cues; however, it tends to produce false detections under complex backgrounds or low-contrast conditions. Cong et al. [10] introduced a global–local collaborative learning approach, yet its performance degrades under low-light conditions due to poor depth map quality. Zhao et al. [11] constructed an edge-aware network that enhances saliency features through multi-scale feature aggregation, though it remains unstable when dealing with low-quality depth inputs.

In addition, several studies have explored incorporating edge information to guide models in detecting salient objects with well-defined boundaries. Deng et al. [12] proposed a recursive residual optimization-based SOD method, which progressively refines saliency maps through recursive residual modules, enhancing boundary and detail preservation. By combining boundary and pixel-level losses with iterative optimization, this method improves fine-grained object detection accuracy, though it still struggles with highly complex scenes and intricate textures. Liu et al. [13] introduced a pooling-based lightweight network design aimed at real-time SOD applications. Through multi-scale pooling operations, the model achieves a favorable balance between accuracy and computational efficiency, making it suitable for real-time scenarios; however, it exhibits limitations in capturing fine-grained details in complex environments.

### 2.2. Salient Object Detection in Optical Remote Sensing Images

In recent years, salient object detection in optical remote sensing images has become an active research topic. Researchers have predominantly employed convolutional neural network (CNN)-based approaches to model and extract salient features from remote sensing imagery, achieving promising detection performance. For instance, Li et al. [14] proposed a U-Net-based multi-resolution feature inference method that effectively integrates features across different scales to improve detection accuracy; however, its adaptability remains limited when handling targets with significant morphological variations. Subsequently, Li et al. [15] designed a network comprising five closely connected parallel paths, enabling comprehensive utilization of both intra-path and inter-path information to enhance feature representation capability. Nevertheless, its high computational complexity poses challenges for real-time applications. Zhang and Ma [16] further combined a convolutional encoder–decoder architecture with a recurrent neural network (RNN), thereby enhancing global contextual modeling and improving fine-grained feature representation; however, the method still exhibits deficiencies in handling complex background interference and fine-detail feature processing.

The ORSSD dataset [17] was constructed and the LVNet model was developed to enhance salient object detection performance. In LVNet, an L-shaped dual-stream pyramid module is employed to extract multi-scale features, while a V-shaped nested encoder–decoder structure is adopted to highlight salient objects and suppress background interference. However, the effectiveness of multi-scale feature fusion and background suppression is constrained by high background complexity. Zhang et al. [18] extended the EORSSD dataset by introducing edge supervision [19] and proposed DAFNet, which achieves shallow-to-deep attention information transmission while simultaneously predicting saliency and edge maps to enhance boundary representation. Although this approach improves detection performance through edge-aware learning, it remains prone to boundary–object confusion under highly complex or dynamically changing backgrounds, leading to instability in saliency map generation. Huang et al. [20] integrated high-level features to localize salient objects and utilized them to guide shallow feature refinement for coarse saliency map generation. However, due to the insufficient detail-capturing capacity of high-level features—particularly around object boundaries or in cluttered backgrounds—the accuracy of saliency maps may degrade. Li et al. [21] further explored the complementarity among foreground, background, and global image-level information, integrating them to enhance discriminability in RSI-SOD tasks.The LVNet proposed by Li et al. [3], based on the ORSSD dataset, effectively improves target prominence through multi-scale feature fusion and background suppression but still struggles under complex background conditions. To address insufficient edge detection, DAFNet [18] introduced edge supervision on the EORSSD dataset, improving boundary delineation yet remaining susceptible to confusion between edges and salient regions in dynamic scenes. Huang et al. [20] attempted to refine shallow features guided by high-level semantics, which enhanced overall detection but reduced boundary precision due to limited fine-detail representation. Li et al. [22] proposed a unified modeling framework combining foreground, background, and global cues to strengthen saliency discrimination. Zhou et al. [8] emphasized the integration of edge awareness and multi-resolution features, though its dependence on clear boundaries limits performance in low-resolution imagery. Some studies, such as Li et al. [20], explored inter-branch contextual information transfer to enhance feature representation, yet excessive reliance on contextual exchange may compromise focus on salient regions. With the introduction of Transformer-based architectures [13], global modeling capability has been substantially improved; however, their high computational cost and low inference efficiency introduce new challenges. Meanwhile, weakly supervised methods utilizing Grad-CAM to generate pseudo-labels have reduced manual annotation costs, but the limited precision of pseudo-labels still negatively impacts performance.Ma et al. [23] combined scribble-based re-annotation with a multi-branch edge detection network to refine boundary localization, achieving notable improvements. Additionally, lightweight models such as FSMINet [24] and SeaNet [25] enhance real-time performance through structural simplification, albeit with some loss in detection accuracy for high-precision tasks [26].

## 3. Hierarchical Semantic Interaction-Based Salient Object Detection Method for Remote Sensing Images

### 3.1. Network Architecture Design

**Figure 1:**
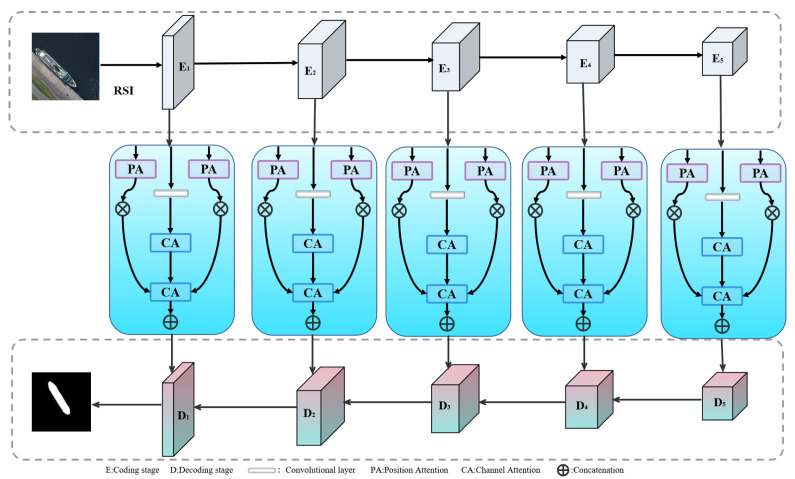
HSIMNet model network structure diagram.

In this work, we propose a novel salient object detection architecture named HSIMNet, specifically designed for optical remote sensing images. The overall framework follows an encoder–decoder structure, as illustrated in Figure 1. HSIMNet primarily consists of three original components: an encoder network, a Hierarchical Semantic Interaction Module (HSIM), and a decoder network. Unlike existing architectures, HSIMNet focuses on hierarchical semantic interaction and multi-scale complementary feature aggregation to enhance saliency perception and object structure preservation.

The encoder network is constructed based on a modified VGG-16 backbone, which is adapted to better capture multi-level semantic and spatial cues from remote sensing imagery. This provides a strong feature foundation for handling complex scenes that typically exhibit heterogeneous content and weak object boundaries.

The core contribution of our framework lies in the Hierarchical Semantic Interaction Module, which we propose to address the limitations of insufficient global understanding and poor boundary awareness in existing SOD models. The module generates multi-scale complementary saliency features by jointly modeling foreground–background contrast, edge structure information, and global contextual semantics. Through hierarchical interaction, the module progressively enhances feature discriminability and significantly improves the representation of small and low-contrast salient targets.

The decoder network subsequently reconstructs high-resolution saliency maps using progressive upsampling and multi-level feature fusion. By aligning semantic and spatial information at different stages, the decoder enables fine-grained delineation of object boundaries and accurate pixel-level localization.

Overall, HSIMNet introduces a complete architectural pipeline that integrates hierarchical interaction, semantic complementarity, and structural restoration, effectively improving robustness and detection accuracy across diverse remote sensing environments.

### 3.2. Hierarchical Semantic Interaction Module

To enhance cross-level semantic interaction and improve feature discrimination under complex backgrounds in optical remote sensing images, we propose a Hierarchical Semantic Interaction Module (HSIM). Unlike traditional SOD methods that rely solely on foreground priors or independent attention mechanisms for highlighting salient regions, the proposed HSIM performs hierarchical semantic guidance and dual-attention fusion to jointly utilize foreground, background, and boundary cues, enabling more accurate salient object perception.

As shown in Figure 1, the HSIM receives multi-scale features from the backbone network and extracts fine-grained semantic representation through a hierarchical interaction structure. Conventional convolution-based feature extraction strategies are limited in capturing the correlations between channel-wise semantic information and spatial structural cues. To overcome this limitation, the proposed module integrates a Channel Attention Mechanism CAM [24] and a Position Attention Mechanism (PAM) [25] to effectively model global channel dependencies and spatial positional relationships, respectively. This design enhances the perception of salient targets while suppressing background interference [26].

The processing pipeline of the HSIM is summarized as follows: the backbone first generates initial feature maps, which are fed into the CAM to adaptively recalibrate channel responses based on global semantic dependenciess [27]. Meanwhile, the PAM highlights spatially important regions by encoding pixel-wise relationships across the feature map. Finally, the outputs of the CAM and PAM branches are fused to produce a content-enhanced multi-dimensional semantic feature representation, enabling more precise detection of salient objects with clear boundaries and robust context understanding.

**Figure 2:**
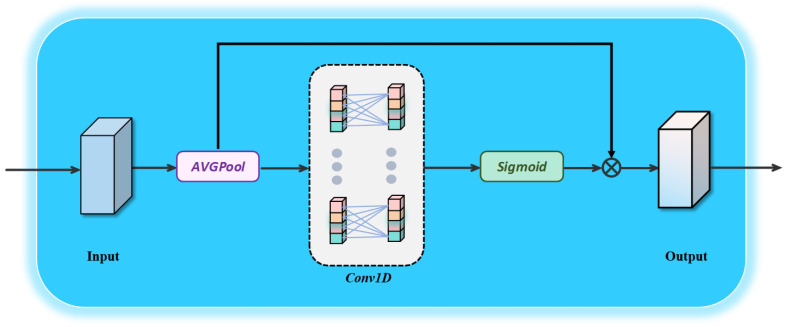
Channel Attention Mechanism diagram.

The detailed process of the Channel Adaptive Enhancement Module (CAE) [28] is illustrated in Figure 2. Within the Hierarchical Semantic Interaction Module, the primary purpose of the CAE is to model inter-channel relationships globally by adaptively weighting feature maps across different channels. This enables the network to better capture the associations and dependencies among various regions of the image, thereby effectively identifying salient regions in optical remote sensing images characterized by complex backgrounds and diverse object types. The process of the Channel Adaptive Enhancement Module includes the following steps:

(1) Channel-wise Global Average Pooling: The input feature map undergoes Global Average Pooling (GAP) to compute a global feature descriptor for each channel. The specific formulation is as follows:

(1) $$GAP\left(F_{t}^{e}\right)=\frac{1}{W\times H}\sum_{i=1}^{H}\sum_{j=1}^{W}F_{t}^{e}\left(i,j\right)$$

where *H* denotes the height of the feature map, and $F_{t}^{e}\left(i,j\right)$ represents the feature value at position (i,j). This operation extracts the global information of each channel without reducing the channel dimensionality.

(2) Channel Weighting: The channel weights are generated by applying a one-dimensional convolution to the globally pooled features $GAP(F_{t}^{e})$. To better capture inter-channel dependencies, a one-dimensional convolution is employed to model the local interactions between each channel and its *k* nearest neighboring channels. The kernel size *k*, a hyperparameter, defines the interaction range for each channel and its local neighborhood. This process can be expressed as follows:

(2) $$F_{t}^{ca}=\sigma \left(Conv_{1}^{k}\left(GAP\left(F_{t}^{e}\right)\right)\right)$$

where $F_{t}^{ca}$ denotes the channel weight, σ represents the Sigmoid activation function, and $Conv_{1}^{k}$ refers to the one-dimensional convolution operation used to capture local cross-channel interactions with a kernel size of *k*.

(3) Relationship Between Channel Dimension and Kernel Size: To adaptively adjust the relationship between kernel size and channel dimension across different network architectures, the following formulation is proposed:

(3) $$C=\psi \left(k\right)=2^{\left(\gamma \times k+b\right)}$$

where *C* denotes the number of channels, *k* is the kernel size, and γ=2, b=1 are parameters controlling the proportional relationship between kernel size and channel dimension.

The kernel size *k* can be inversely derived from the channel dimension *C* as follows:

(4) $$k=\psi \left(C\right)={\left\lfloor \frac{\log_{2}\left(C\right)+b}{\gamma }\right\rfloor }_{odd}$$

where ${\left\lfloor \cdot \right\rfloor }_{odd}$ denotes rounding to the nearest odd integer. This adaptive formulation allows the model to automatically adjust the kernel size according to the channel dimension, enabling more effective inter-channel interaction modeling and achieving an optimal balance between computational efficiency and detection performance.

(4) Feature Weighting: The generated channel weights $F_{t}^{ca}$ are multiplied with the input feature map $F_{t}^{e}$ to obtain the weighted feature map.

**Figure 3:**
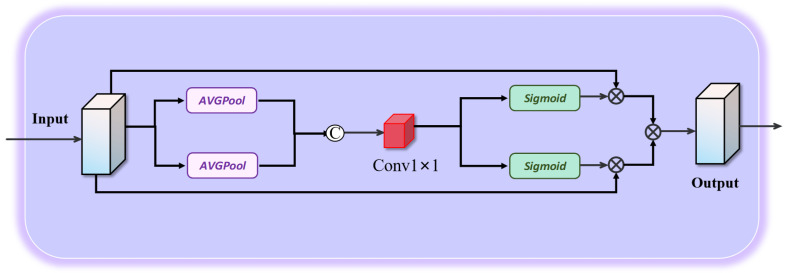
Position attention mechanism structure diagram.

The detailed process of the Position-Aware Spatial Selection Module (PSS) is illustrated in Figure 3. The objective of this module is to enhance the model’s understanding of spatial structural information by assigning adaptive weights to spatial locations, thereby emphasizing the most salient regions. The process involves the following steps:

A. Horizontal and Vertical Pooling: The input feature map E(i,j) is first subjected to pooling operations along the horizontal $p_{w}$ and vertical $p_{h}$ directions to extract spatial structural information. The pooling operations are defined as follows:

(5) $$p_{w}\left(i\right)=\frac{1}{H}\sum_{j=0}^{H}E\left(i,j\right),\;p_{h}\left(j\right)=\frac{1}{W}\sum_{i=0}^{W}E\left(i,j\right)$$

where $p_{w}$ and $p_{h}$ represent the horizontal and vertical features, respectively, and *W* and *H* denote the width and height of the feature map. The pooling operation facilitates the preservation of spatial structural information within the image.

B. Generation of Positional Attention Coordinates: The pooled features from the horizontal and vertical directions are concatenated and processed through a convolution operation to generate the positional attention coordinates. The computation is defined as follows:

(6) $$P\left(a_{w},a_{h}\right)=\mathrm{Conv}\left[\mathrm{Concat}\left(p_{w},p_{h}\right)\right]$$

where Conv denotes the 1×1 convolution operation, and Concat represents the concatenation operation. The resulting positional attention coordinates $P(a_{w},a_{h})$ capture the weighted information of each spatial location.

C. Segmentation of Positional Attention Features: The generated attention coordinates are partitioned to obtain feature maps in the horizontal and vertical directions:

(7) $$s_{w}=\mathrm{Split}\left(a_{w}\right),\;s_{h}=\mathrm{Split}\left(a_{h}\right)$$

where Split denotes the segmentation operation, and $s_{w}$ and $s_{h}$ represent the feature maps in the horizontal and vertical directions, respectively. This step ensures that the Position-Aware Spatial Selection Module can independently extract features along different spatial dimensions, thereby enhancing the model’s spatial representation capability.

D. Final Output: The features from the channel and position-aware spatial selection modules are integrated and weighted to generate the final feature output:

(8) $$F_{CPAM}=E\times s_{w}\times s_{h}$$

where *E* denotes the combined channel and position attention weight matrix, and $F_{CPAM}$ represents the multi-content feature map integrating both channel and spatial information, effectively capturing image details and spatial structural characteristics.

(5) Multi-Content Feature Fusion Module: By integrating the feature maps generated by the Channel Adaptive Enhancement Module and the Position-Aware Spatial Selection Module, a multi-level feature representation of the image is obtained. These feature maps jointly capture both channel-wise and spatial positional information. The final feature fusion is formulated as follows:

(9) $$F_{final}=F_{t}^{ca}⊙F_{CPAM}$$

In this manner, the Hierarchical Semantic Interaction Module fully leverages the strengths of both the channel and position-aware spatial selection modules, thereby enhancing its performance in complex tasks.

### 3.3. Loss Function

The Hierarchical Semantic Interaction Module effectively exploits the advantages of both the channel and position-aware spatial selection modules, thereby enhancing performance in complex tasks. To develop a successful CNN-based model, in addition to an efficient architecture and well-designed modules, an effective training strategy can further improve model performance without increasing parameter complexity. During training, deep supervision is employed to monitor intermediate saliency maps of varying scales, encouraging the network to learn multi-scale salient features. Moreover, inspired by the successful application of hybrid and complementary losses in salient object detection, the loss function integrates the classical pixel-level cross-entropy loss, the feature map-level IoU loss, and a metric-aware F-measure loss to further facilitate network optimization. Consequently, a comprehensive loss function $L_{t}^{s}$ is formulated to supervise the predicted saliency map $S_{t}$, defined as follows:

(10) $$L_{t}^{s}=l_{bce}\left(up\left(S_{t}\right),G\right)+l_{iou}\left(up\left(S_{t}\right),G\right)+l_{fm}\left(up\left(S_{t}\right),G\right)$$

where *G* denotes the ground truth, and $l_{bce}\left(\cdot \right)$, $l_{iou}\left(\cdot \right)$, and $l_{fm}\left(\cdot \right)$ represent the BCE loss, IoU loss, and F-measure loss, respectively. Their formulations are as follows:

(11) $$l_{bce}=-\sum_{i=1}^{W\times H}\left[G\left(i\right)\log\left(S\left(i\right)\right)+\left(1-G\left(i\right)\right)\log\left(1-S\left(i\right)\right)\right]$$

(12) $$l_{iou}=1-\frac{\sum_{i=1}^{W\times H}S\left(i\right)\cdot G\left(i\right)}{\sum_{i=1}^{W\times H}\left[S\left(i\right)+G\left(i\right)-S\left(i\right)\cdot G\left(i\right)\right]}$$

(13) $$l_{fm}=1-\frac{\left(1+\beta^{2}\right)\cdot P\left(S,G\right)\cdot R\left(S,G\right)}{\beta^{2}\cdot P\left(S,G\right)+R\left(S,G\right)}$$

where G(i)∈{0,1} and S(i)∈[0,1] denote the ground truth label and the predicted saliency score of the *i* pixel, respectively. The parameter $\beta^{2}$ is set to 0.3.

Precision (P) and Recall (R) are defined as follows:

(14) $$\mathrm{Precision}\;\left(P\right)=\frac{TP}{TP+FP}$$

(15) $$\mathrm{Recall}\;\left(R\right)=\frac{TP}{TP+FN}$$

where $TP\left(S,G\right)=\sum_{i=1}^{W\times H}S\left(i\right)\cdot G\left(i\right)$, $FP\left(S,G\right)=\sum_{i=1}^{W\times H}S\left(i\right)\left(1-G\left(i\right)\right)$, and $FN\left(S,G\right)=\sum_{i=1}^{W\times H}\left(1-S\left(i\right)\right)G\left(i\right)$.

Additionally, an auxiliary model is employed to learn and generate the edge map $a_{t}^{e}$, which is trained by minimizing the edge loss $L_{t}^{e}$, defined as follows:

(16) $$L_{t}^{e}={∥a_{t}^{e}-{\hat{a}}_{t}^{e}∥}_{2}^{2},$$

where $G_{e}$ denotes the ground truth edge map, generated following the method described in [17]. Finally, the total loss of the network during training, $L_{total}$, is defined as follows:

(17) $$L_{total}=\sum_{t=1}^{5}\left(L_{t}^{s}+L_{t}^{e}\right)$$

## 4. Experiments

### 4.1. Datasets

The experiments in this study are conducted on two publicly available remote sensing salient object detection datasets: ORSSD [2] and EORSSD [5]. ORSSD contains 800 optical remote sensing images with pixel-level manually annotated saliency masks, including 600 images for training and 200 for testing. EORSSD, as an extended version of ORSSD, consisting of 2000 images, divided into 1400 for training and 600 for testing.

Both datasets include diverse and challenging real-world remote sensing scenes, such as strong illumination, shadow occlusion, cloud interference, land–sea boundaries, and complex background textures. These conditions pose significant difficulty for salient object detection tasks and provide a comprehensive benchmark for evaluating model robustness and generalization. In our experiments, all images and ground-truth masks are used in their original resolutions, and standard data-augmentation strategies (random flipping, rotation, and cropping) are applied during training to enhance model stability and mitigate overfitting.

### 4.2. Implementation Details

The model is implemented using the PyTorch framework (version 1.13.1), and all experiments are conducted on a workstation equipped with an Intel Core i9-9900X @ 3.50 GHz CPU, 32 GB RAM, and an NVIDIA GTX 3090 GPU. The proposed HSIMNet model supports end-to-end training and employs the Adam optimizer for parameter updates.

To configure the training hyperparameters of RSI-SOD, we performed systematic empirical tuning, taking into account the characteristics of high-resolution remote sensing imagery. The initial learning rate ($1.0\times 10^{-4}$) was chosen based on preliminary experiments and common practices for salient object detection networks using the Adam optimizer. We evaluated several candidate learning rates in the range of $5\times 10^{-5}$ to $5\times 10^{-4}$ and found that $1.0\times 10^{-4}$ produced the most stable optimization behavior while avoiding gradient explosion and oscillations common in complex remote sensing scenes.

Since the multi-scale fusion architecture of HSIMNet leads to high GPU memory consumption, we set the batch size to 4. Experiments with batch sizes of 2, 4, and 8 showed that a batch size of 4 achieved a good balance between training stability and GPU utilization, while a batch size of 8 exceeded the memory capacity of an RTX 3090 GPU.

The maximum number of iterations was determined based on convergence analysis. During training, both the MAE and F-value curves stabilized before 120 iterations, with minimal improvement thereafter. Therefore, we set the maximum number of iterations to 120 to ensure efficient training and avoid unnecessary computational costs.

Finally, the decay step size of 40 in the learning rate scheduling was derived through validation experiments. A decay interval of 40 epochs ensures that the learning rate decreases at an appropriate rate, preventing overfitting in the later stages of training while maintaining sufficient learning capacity to handle the complex spatial structures in optical remote sensing imagery.

### 4.3. Quantitative Comparison

To validate the effectiveness of the proposed network, it is compared with 12 state-of-the-art methods.

The proposed model is evaluated against SOD methods designed for both NSI and ORSI scenarios, including CorrNet [13], DAFNet [18], EGNet [11], EMFINet [29], F3Net [30], GateNet [31], LVNet [17], MJRBM [32], R3Net [12], SARNet [2], U2Net [33], and SUCA [34]. For fair comparison, the results of all benchmark methods were derived from the saliency maps publicly released by the original authors on the ORSSD and EORSSD datasets. This approach minimizes the impact of parameter configuration differences on the comparison results. This ensures the comparability and accuracy of the overall evaluation. These methods are standard benchmarks in the field of remote sensing SOD [26].

Table 1 presents the quantitative results for the four evaluation metrics: Sm, MAE, maxE, and max F. It is noteworthy that lower MAE values indicate better performance, whereas higher values for the other three metrics correspond to superior results. Compared with the 12 competing methods, the proposed approach achieves outstanding performance across all four metrics, surpassing nearly all existing methods, with DAFNet being the closest competitor. Specifically, the proposed method attains the best MAE value on the ORSSD dataset and demonstrates strong performance on both the MAE and maxF metrics, with the maxF score exceeding the second-best result by 0.0156. Moreover, the method achieves a notable improvement in the Sm metric, reaching 0.94415, and it attains competitive performance in the maxE metric, ranking second only to DAFNet.

On the EORSSD dataset, the proposed method achieves the best performance in both Sm and maxF metrics, with scores of 0.9205 and 0.9100, respectively. The MAE and maxE values rank second, and although slight differences exist between the proposed method and DAFNet in these metrics, the discrepancies are not statistically significant.

**Figure 4:**
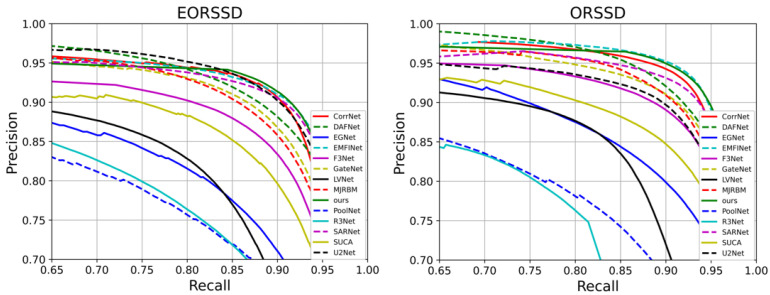
Precision–Recall (PR) curves of the proposed HSIMNet and comparison methods on the ORSSD and EORSSD datasets.

To comprehensively evaluate the strengths and weaknesses of each comparative method, multiple dimensions of analysis are conducted, including the quantitative results presented in Table 1 and corresponding visualizations. Figure 4 illustrates the PR curves of all methods, where curves closer to the top-right corner indicate superior performance. The results demonstrate that HSIMNet outperforms all competing approaches on both the EORSSD and ORSSD datasets.

**Figure 5:**
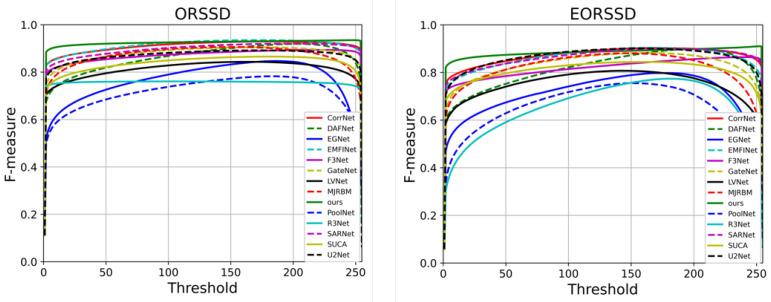
F-measure curves of HSIMNet and other state-of-the-art methods on the ORSSD and EORSSD datasets.

Furthermore, to provide a more detailed comparison, Figure 5 presents the F-measure curves, where larger enclosed areas correspond to better performance. It can be observed that HSIMNet achieves superior results on both the PR and F-measure curves—the PR curve lies closest to the coordinate point (1, 1), while the F-measure curve encloses the largest area, confirming the model’s overall advantage.

### 4.4. Qualitative Comparison

**Figure 6:**
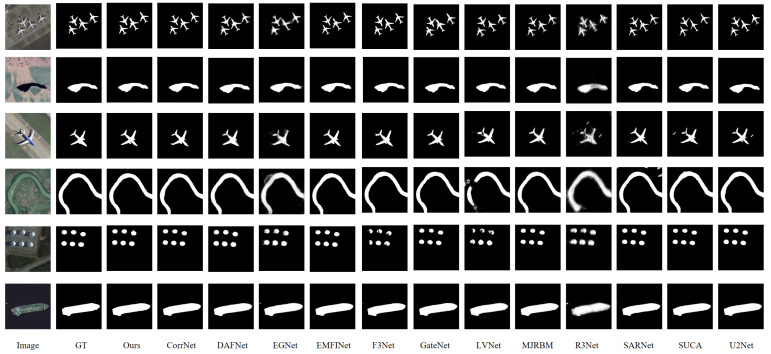
Qualitative comparison of salient object detection results produced by HSIMNet and representative competing methods on challenging scenes from the ORSSD and EORSSD datasets.

Figure 6 presents the qualitative visual comparison of various saliency models applied to optical remote sensing images containing targets such as lakes, roads, and both large and small buildings. All models, including HSIMNet and other deep learning-based approaches, were trained on the same datasets. By accurately localizing and segmenting salient objects in complex and challenging scenes, HSIMNet demonstrates superior overall visual performance compared with the other models.

To more intuitively demonstrate the advantages of HSIMNet, several challenging test samples were selected for comparative analysis. The first column shows the original images, the second displays the corresponding ground truth, the third presents the results of HSIMNet, and the remaining columns depict the outputs of other state-of-the-art salient object detection (SOD) methods. As illustrated in Figure 6, HSIMNet exhibits exceptional robustness in handling globally salient targets, primarily attributed to the Hierarchical Semantic Interaction Module, which effectively extracts semantic features of optical remote sensing images from multiple perspectives and scales.

Furthermore, Figure 6 includes several small-scale salient targets, which pose a significant challenge in optical remote sensing saliency detection. HSIMNet accurately captures the shapes of these smaller salient objects and generates fine-grained saliency maps. For instance, in the fourth row of Figure 6, which depicts a scene containing large buildings, most methods are affected by a narrow road outside the salient region that shares a similar color with the boundary. In contrast, HSIMNet precisely delineates the boundary of the salient region, effectively mitigating interference and accurately localizing the target area. These results further demonstrate the strong saliency detection capability and robustness of HSIMNet across diverse scenarios.

### 4.5. Ablation Study

In this section, the effectiveness of different modules within the proposed network is analyzed through a series of ablation experiments. Specifically, the Channel Adaptive Enhancement Module and the Position-Aware Spatial Selection Module within the Hierarchical Semantic Interaction Module are examined. To evaluate the contribution of each component, a U-shaped encoder–decoder network with skip connections—excluding the Hierarchical Semantic Interaction Module—is adopted as the baseline model. Subsequently, the individual contributions of each component within the proposed framework are investigated, including the Channel Adaptive Enhancement Module and the Position-Aware Spatial Selection Module. The quantitative results for various module combinations are presented in Table 2 and Table 3.

As shown in Table 2 (EORSSD) and Table 3 (ORSSD), the model’s performance metrics (maxF, maxE, and Sm) consistently improve with the progressive integration of the key modules (CA and PA). This indicates that each module independently enhances performance to a certain extent, while their combined effect further strengthens overall model capability. When the CA module is introduced individually, a noticeable improvement is observed, demonstrating its effectiveness in enhancing the model’s ability to capture global contextual information and refine saliency detection. Similarly, the inclusion of the PA module yields significant performance gains—particularly on the EORSSD dataset, where the improvements in maxF and Sm are especially prominent—highlighting its strong contribution to fine-grained local feature representation and small-object detection. When both modules are integrated, the model achieves its best overall performance, confirming that the CA and PA modules provide complementary benefits through mutual reinforcement, thereby strengthening both global and local feature extraction capabilities. The baseline model alone struggles to effectively capture global and detailed salient features. With the integration of the CA and PA modules, maxF, maxE, and Sm increase by 0.0274, 0.0184, and 0.0189, respectively, on the EORSSD dataset—an evident improvement that demonstrates the modules’ effectiveness in handling complex scenes featuring diverse salient targets such as lakes and roads. On the ORSSD dataset, the addition of the CA and PA modules results in performance gains of 0.0138, 0.0056, and 0.0123 in maxF, maxE, and Sm, respectively. Although the improvement margin is smaller than that on EORSSD, the enhancement reflects a more refined optimization, indicating the model’s capacity for precision-oriented saliency detection. Overall, these findings confirm that the inclusion of the CA and PA modules significantly enhances model performance, validating the effectiveness of the proposed module design. The greater improvement observed on the EORSSD dataset underscores the modules’ robustness in handling complex environments, while the refined gains on ORSSD highlight the model’s superior precision and adaptability across both complex and simpler scenarios.

We performed an ablation study to evaluate the contribution of each loss term (Table 4). Using only CE loss results in lower performance (max F = 0.8750, maxE = 0.9550, S-measure = 0.8950). Adding IoU loss or F-measure loss individually improves spatial consistency and metric alignment. The combination of all three losses achieves the best results (max F = 0.9100, maxE = 0.9727, S-measure = 0.9205), demonstrating that each component contributes complementary information and justifying the hybrid loss design.

### 4.6. Limitations and Future Work

Although the proposed HSIMNet achieves state-of-the-art performance on multiple optical remote sensing salient object detection datasets, several limitations remain. First, the model is trained on datasets that may not fully cover the diversity of real-world remote sensing conditions, such as extreme illumination variations, haze, dense cloud coverage, or sensor-dependent radiometric differences. As a result, the model’s generalization to unseen sensors or complex environmental conditions may be limited. Second, while the hierarchical spectral–spatial interaction modules effectively capture multi-level features, HSIMNet may still struggle with very small or low-contrast salient objects, or in scenarios with highly cluttered backgrounds, due to feature loss in deeper layers. Third, the computational cost during training is relatively high because of the multi-branch interaction structure and high-resolution input. Although inference is efficient on high-end GPUs, deploying HSIMNet on low-resource or edge devices may require model compression, pruning, or knowledge distillation techniques. In future work, we plan to address these limitations by:

(1) Extending the training datasets to include more diverse and challenging remote sensing scenarios to improve generalization.

(2) Exploring adaptive multi-scale feature enhancement and attention mechanisms to better capture very small or low-contrast salient objects.

(3) Developing lightweight and resource-efficient variants of HSIMNet to enable deployment on edge platforms while maintaining competitive accuracy.

## 5. Conclusions

This study proposes a Hierarchical Semantic Interaction Network (HSIMNet) for salient object detection in optical remote sensing images [26]. To address challenges such as complex background interference and diverse target topologies, HSIMNet introduces a Hierarchical Semantic Interaction Module (HISM), which enhances saliency representation through multi-scale complementary feature extraction, thereby improving both accuracy and robustness. Built upon the VGG-16 backbone, the model retains the first five convolutional blocks for feature extraction and integrates the Hierarchical Semantic Interaction Module to achieve collaborative modeling of foreground, background, and edge information. Additionally, the incorporation of the Channel Adaptive Enhancement Module (CAM) and the Position-Aware Spatial Selection Module (PAM) further strengthens feature representation capability. In the decoding stage, the model progressively restores spatial resolution through deconvolution layers and performs multi-level feature fusion. During training, a composite loss function is employed to jointly optimize the network, comprehensively enhancing saliency detection performance. Overall, HSIMNet demonstrates superior robustness and high precision under complex background and low-contrast conditions, providing an efficient and reliable solution for salient object detection in optical remote sensing images.

## Figures and Tables

**Table 1:** Performance indicators of different methods on two datasets.

Method	ORSSD									EORSSD								
	M↓	$F_{\beta }^{\max}↑$	$F_{\beta }^{\mathrm{mean}}↑$	$F_{\beta }^{\mathrm{adp}}↑$	$F_{\xi }^{\max}↑$	$F_{\xi }^{\mathrm{mean}}↑$	$F_{\xi }^{\mathrm{adp}}↑$	$S_{\alpha }↑$	$F_{\beta }^{w}↑$	M↓	$F_{\beta }^{\max}↑$	$F_{\beta }^{\mathrm{mean}}↑$	$F_{\beta }^{\mathrm{adp}}↑$	$F_{\xi }^{\max}↑$	$F_{\xi }^{\mathrm{mean}}↑$	$F_{\xi }^{\mathrm{adp}}↑$	$S_{\alpha }↑$	$F_{\beta }^{w}↑$
CorrNet	0.0107	0.9293	0.9077	0.8635	0.9789	0.9687	0.9602	0.9405	0.8989	0.0087	0.9025	0.8690	0.7828	0.9695	0.9512	0.9281	0.9297	0.8615
DAFNet	0.0111	0.9192	0.8578	0.7601	0.9822	0.9496	0.8836	0.9186	0.8274	0.0060	0.8985	0.8135	0.6502	**0.9805**	0.9295	0.8043	0.9175	0.7812
EGNet	0.0217	0.8468	0.7603	0.6584	0.9720	0.8959	0.8226	0.8725	0.7039	0.0111	0.7977	0.7054	0.5541	0.9398	0.8621	0.7561	0.8599	0.6614
EMFINet	0.0104	0.9351	0.9083	0.8565	0.9813	0.9662	0.9577	0.9435	0.9028	0.0079	0.9036	0.8601	0.7555	0.9703	0.9445	0.9126	**0.9307**	0.8497
F3Net	0.0155	0.8939	0.8677	0.8240	0.9651	0.9538	0.9399	0.9160	0.8485	0.0088	0.8644	0.8164	0.7428	0.9579	0.9401	0.9072	0.9030	0.7987
GateNet	0.0144	0.9049	0.8769	0.8163	0.9661	0.9472	0.9180	0.9192	0.8600	0.0099	0.8822	0.8314	0.6955	0.9594	0.9238	0.8504	0.9090	0.8136
LVNet	0.0211	0.8434	0.8107	0.7512	0.9454	0.9204	0.9106	0.8807	0.7867	0.0146	0.8071	0.7488	0.6308	0.9274	0.8729	0.8323	0.8639	0.7219
MJRBM	0.0151	0.9061	0.8679	0.7959	0.9681	0.9338	0.9196	0.9190	0.8545	0.0105	0.8807	0.8162	0.6754	0.9638	0.9087	0.8506	0.9068	0.8030
R3Net	0.0399	0.7608	0.7529	0.7529	0.8903	0.8674	0.8687	0.8143	0.7400	0.0173	0.7738	0.6521	0.4298	0.9496	0.8315	0.6470	0.8191	0.5489
SARNet	0.0105	0.9202	0.8942	0.8473	0.9702	0.9596	0.9305	0.9361	0.8821	0.0091	0.8995	0.8625	0.7716	0.9662	0.9482	0.8879	0.9286	0.8522
SUCA	0.0150	0.8655	0.8347	0.7755	0.9582	0.9349	0.8963	0.8992	0.8106	0.0102	0.8441	0.8040	0.7111	0.9487	0.9131	0.8569	0.8971	0.7942
U2Net	0.0166	0.8917	0.8669	0.8202	0.9539	0.9386	0.9326	0.9156	0.8551	**0.0073**	0.9008	0.8603	0.7462	0.9645	0.9370	0.8993	0.9218	0.8517
Ours	**0.0101**	**0.9348**	**0.9194**	**0.9061**	**0.9804**	**0.9741**	**0.9773**	**0.9441**	**0.9151**	0.0079	**0.9100**	**0.8792**	**0.8400**	**0.9727**	**0.9645**	**0.9542**	0.9295	**0.8751**

**Table 2:** Performance indicators of different modules on EORSSD.

Baseline	CA	PA	max F ↑	maxE ↑	Sm ↑
√			0.8826	0.9603	0.9026
√	√		0.8955	0.9688	0.9151
√		√	0.8994	0.9695	0.9168
√	√	√	0.9100	0.9727	0.9295

**Table 3:** Performance indicators of different modules on ORSSD.

Baseline	CA	PA	max F ↑	maxE ↑	Sm ↑
√			0.9208	0.9738	0.9328
√	√		0.9216	0.9759	0.9385
√		√	0.9258	0.9766	0.9396
√	√	√	0.9348	0.9804	0.9441

**Table 4:** Ablation study on the contribution of each loss component on EORSSD.

CE	IoU	F-Measure	max F ↑	maxE ↑	S_m ↑
√			0.8750	0.9550	0.8950
√	√		0.8880	0.9630	0.9100
√		√	0.8920	0.9650	0.9120
√	√	√	0.9100	0.9727	0.9205

## Data Availability

The datasets analyzed in this study are publicly available. The ORSSD dataset can be accessed at https://github.com/rmcong/ORSSD-dataset (accessed on 10 September 2025), and the EORSSD dataset at https://github.com/rmcong/EORSSD-dataset (accessed on 14 September 2025). No new data were created or analyzed in this study.

## Abbreviations

**Abbreviation**	**Full Name**
SOD	Salient Object Detection
RSI	Remote Sensing Image
HSIM	Hierarchical Semantic Interaction Module
CAM	Channel Attention Mechanism
PAM	Position Attention Mechanism
CNN	Convolutional Neural Network
MAE	Mean Absolute Error
EORSSD	Extended Optical Remote Sensing Salient Object Detection Dataset
ORSSD	Optical Remote Sensing Salient Object Detection Dataset
